# Glial Cell Activation and Immune Responses in Glaucoma: A Systematic Review of Human Postmortem Studies of the Retina and Optic Nerve

**DOI:** 10.14336/AD.2024.0103

**Published:** 2024-10-01

**Authors:** Akanksha Salkar, Roshana Vander Wall, Devaraj Basavarajappa, Nitin Chitranshi, Gabriella E. Parilla, Mehdi Mirzaei, Peng Yan, Stuart Graham, Yuyi You

**Affiliations:** ^1^Department of Clinical Medicine, Faculty of Human, Health, and Medical Science, Macquarie University. Sydney, NSW, Australia.; ^2^Save Sight Institute, University of Sydney. Sydney, NSW, Australia.; ^3^Department of Ophthalmology & Vision Sciences, University of Toronto, Kensington Eye Institute/UHN, Canada.

**Keywords:** Glaucoma, POAG, post-mortem, human, retina, Optic nerve head

## Abstract

Although researched extensively the understanding regarding mechanisms underlying glaucoma pathogenesis remains limited. Further, the exact mechanism behind neuronal death remains elusive. The role of neuroinflammation in retinal ganglion cell (RGC) death has been prominently theorised. This review provides a comprehensive summary of neuroinflammatory responses in glaucoma. A systematic search of Medline and Embase for articles published up to 8th March 2023 yielded 32 studies using post-mortem tissues from glaucoma patients. The raw data were extracted from tables and text to calculate the standardized mean differences (SMDs). These studies utilized post-mortem tissues from glaucoma patients, totalling 490 samples, compared with 380 control samples. Among the included studies, 27 reported glial cell activation based on changes to cellular morphology and molecular staining. Molecular changes were predominantly attributed to astrocytes (62.5%) and microglia (15.6%), with some involvement of Muller cells. These glial cell changes included amoeboid microglial cells with increased CD45 or HLA-DR intensity and hypertrophied astrocytes with increased glial fibrillary acidic protein labelling. Further, changes to extracellular matrix proteins like collagen, galectin, and tenascin-C suggested glial cells’ influence on structural changes in the optic nerve head. The activation of DAMPs-driven immune response and the classical complement cascade was reported and found to be associated with activated glial cells in glaucomatous tissue. Increased pro-inflammatory markers such as interleukin-6 (IL-6) and tumor necrosis factor-alpha (TNF-α) were also linked to glial cells. Glial cell activation was also associated with mitochondrial, vascular, metabolic and antioxidant component disruptions. Association of the activated glial cells with pro-inflammatory responses, dysregulation of homeostatic components and antigen presentation indicates that glial cell responses influence glaucoma progression. However, the exact mechanism triggering these responses and underlying interactions remains unexplored. This necessitates further research using human samples for an increased understanding of the precise role of neuroinflammation in glaucoma progression.

## INTRODUCTION

Glaucoma is a neurodegenerative disease characterized by progressive loss of retinal ganglion cells (RGCs), resulting in visual field loss and the optic nerve head (ONH) cupping. This complex neurodegenerative condition is influenced by multiple factors, with advanced age and elevated intraocular pressure (IOP) being prominent risk factors [1]. Unfortunately, it remains one of the leading causes of blindness worldwide and has been estimated to affect approximately 111.8 million individuals by 2040 [2]. Existing literature suggests that glaucoma aetiology is driven by dysregulation of multiple pathways such as biomechanical, vascular, metabolic, oxidative, and inflammatory pathways. This has increased interest in understanding the role of neuroinflammation in glaucoma progression. Neuroinflammation is defined as the microglial or, in certain cases, astrocytic response in the central nervous system (CNS) without any leukocyte infiltration as seen in the non-neural tissues. Since the eye is an extension of the CNS, glaucoma-driven glial response can be defined as neuroinflammation. The precise relationship between neuroinflammation and RGC loss in glaucoma remains an enigma.

Most of the studies focused on neuroinflammatory changes in the retina and ONH have been performed using *in vitro systems* or animal models. However, studies utilizing post-mortem human samples to investigate glaucoma pathology are scarce, likely due to the difficulty in acquiring human samples. The more accessible tissues such as blood, tear film, or even aqueous humour hold the potential for identifying biomarkers. With the existing technologies, primary sites affected by glaucoma—the RGCs and ONH, are not readily accessible for *in vivo* sampling or cellular imaging. Moreover, neuro-inflammation may affect synaptic disruptions within the retina [3]. Therefore, we report the findings of a comprehensive systematic review that investigates the pathological alterations in glaucomatous retina and ONH using post-mortem human samples. Our primary emphasis is on elucidating the neuroinflammatory changes associated with the disease.

### MATERIALS AND METHODS

#### Literature search strategy

The systematic search was conducted in Medline and Embase using the OVID database covering articles published up to 6^th^ March 2023. The search protocol was developed based on Preferred Reporting Items for Systematic Reviews and Meta-Analyses (PRISMA) and World Health Organization (WHO) Review Protocol Template Guidelines where applicable for a systematic review of descriptive (non-interventional) data and is provided in the Supplementary File. A detailed table of the query and search results are provided in the Supplementary File. We also reviewed the bibliography of selected studies to search for more papers fitting the inclusion criteria.

**Figure 1.**
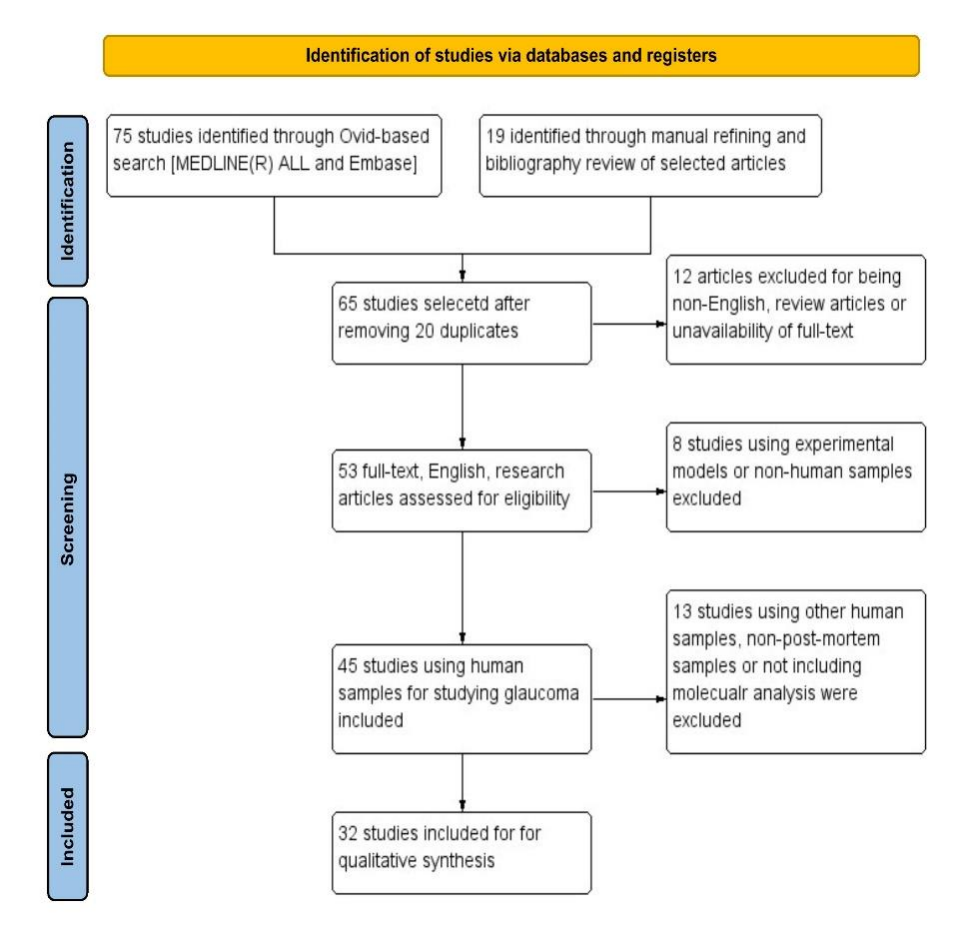
Flow chart of the systematic review. The schematic illustrates the screening process for the review. It could be broadly categorized into identification: using the database and manual review to identify the studies, screening: filtering the studies based on the inclusion criteria and inclusion: final studies included in the review for systematic analysis.

**Table 1:** Overview of the results of all the studies included in the review.

Study	Glaucoma (n)	Control (n)	Age of glaucoma patients (mean ± SD)	Biochemical method	Sampling sites	Markers	Cellular localization	Results
Hernandez et al. 1994	7	9	N/A	ISH and RT-PCR	ONH	Collagen type IV	Astrocytes	↑ in the LC sections and produced by astrocyte
Neufeld et al. 1997	8	6	79±12.3	IHC	ONH	COX-1 and COX-2	Astrocytes	↑COX-1 in astrocytes; Faint COX-2 staining
Pena et al. 1999	15	11	76.1±12.6	IF	ONH	Tenascin C	Astrocytes	↑ of Tenascin in ON, LC, and post-lamina region
Yuan et al. 2000	20	20	79±14	IHC	ONH	TNF-a and TNF-a receptor 1	Astrocytes	↑ TNF-a and TNFR1 parallel to the disease progression
Yan et al. 2000	11	4	78.5±5.05	IHC	ONH	MMPs, TNF-α, and TNF-α receptor 1	Glial cells	↑ the intensity of staining and the number of glial cells.
Tezel et al. 2000	6	6	72.25±10.8	IHC	ONH and Retina	HSP 60 and 27	Astrocytes and RGCs	↑ HSP 60: RGCs and photoreceptors. HSP27: NFL, RGCs, and retinal vessels
Tezel et al. 2001	20	20	78±9	IHC	Retina	TNF-a and TNF-a receptor 1	Astrocytes and RGCs	↑ TNF-a in glial cells and TNF-R1 in the RGCs
Yang et al. 2001	6	6	69.2±14.93	IHC	LC-cross sections	HLA-DR	Astrocytes	↑HLA-DR expression; localised with GFAP positive cells
Yuan et al. 2001	20	20	79 ±17	IHC	ONH	TGF-B1, B2, TNF-α, TNF-R1, COX-1, COX-2, NOS-2, c-fms, MMP-1. MMP-2, MMP-3, MMP14, TIMP1, TIMP2. TSP, CD68, PCNA, HSP27	Microglia	Activated microglia with ↑ in TGF-β2, TNF-α, TNF-R1, COX-1, COX-2, NOS-2, c-fms, MMP-1, TIMP-1, TIMP-2, TSP, CD68, PCNA
Surgucheva et al. 2002	4	6	68.25±4.92	IHC	ON	Synucleins	Glial cells	↑ γ-synuclein in a subset of glial cells in the LC and post-LC regions
Tezel et al. 2003	30	20	80.33±8.98	IHC	Retina	phosphorylated MAPKs	Micro and Macroglia	↑ in p-ERK in glial cells and ↑ p-JNK and p-38 in the RCG layer.
Tezel et al. 2004	28	20	80.4±9.3	IHC	ONH and retina	HIF-1a	RGC and glial cells	↑ HIF-1a in glial cells and RGCs; parallel to the visual defect
Wang et al. 2006	16	10	82.7 ± 9.3	IHC	ONH	Endothelin B receptor	Astrocytes	↑ ETbR in astrocytic processes
Tezel et al. 2007	38	30	76.84±11.06	IHC	ONH and Retina	AGE and RAGE	Astrocytes	↑ extracellular accumulation of AGEs and localization of RAGEs to RGCs and footplates of the Müller cells
Rudzinski et al. 2008	8	8	N/A	RT-PCR and IHC	ONH	VEGF, PDGF and collagen XVIII	Astrocytes	↓VEGF-C and PDGF-A in LC and ↑collagen XVIII and ADAMTSL-3 in LC.
Felichenfeld et al. 2008	5	4	77±9	IHC	ONH	Nitrotyrosine	Astrocytes	↑ Nitrotyrosine in the pre-laminar ONH blood vessels and glial tissue surrounding the pre-laminar ONH
Luo et al. 2010	10	10	84.7 ± 8	IHC and LC-MS/MS	Retina	TLR-2, 3 and 4	Astrocytes and Microglia	LC-MS/MS showed ↑ TLR signalling localised to astrocytes and microglia.
Tezel et al. 2010	38	30	76.8 ± 11	LC-MS and IHC	Retina	CFH, CD35, and CD59.	Whole tissues	Immunolabeling of the complement components and the membrane complex in ILs, including the RGCs and IPLs with regional
Yang et al. 2011	38	30	76.8 ± 11	LC-MS/MS, IHC, and WB	Retina	TNF-a and TNF-a receptor 1	Astrocytes and RGCs	↑ TNFR1-related downstream proteins, regulators of TNFR1 signalling, and TNFAIP3 were upregulated.
Mizokami et al. 2011	1	1	N/A	IHC	ONH	AQP-9 and AQP-4	Glial cells	↑AQP9 co-localized in the glial cells; no change in the AQP4 expression levels
Kerr et al. 2011	2	3	78± 11.3	IHC	ONH and retina	Connexin43	Astrocytes	↑ Connexin43 localized in the LC and near blood vessels and the RGC layer
Reszec et al. 2012	42	2	N/A	IHC	ONH and retina	HIF-1a	Whole tissue	↑ HIF-1a in ON RGCs at the perinuclear and cytoplasmic granular regions.
Goldhagen et al. 2012	10	10	76.5±6.34	IHC	ONH	RhoA, ROCK-1, and ROCK-2	Whole tissue	↑ RhoA, ROCK-1, and ROCK-2 in the ONH localised to prelaminar and laminar regions
Gramlich et al. 2013	6	9	N/A	IF	Retina	IgG accumulation	Whole tissue	IgG deposition in the glaucomatous retina and CD3+/IgG+ plasma cells detected
Funke et al. 2016	4	4	86±9	LC-MS/MS and IHC for validation	Retina	ANT3, MeCp2, DFS70	Whole tissue	LC-MS/MS based analysis of proteome alterations in glaucoma.
Margeta et al. 2018	13	12	79.39±12.90	IHC	ON	CD163 and CD68 for macrophage infiltration	Macrophages	CD163+ macrophages infiltrated the ONH in glaucoma
Belmares et al. 2018	12	12	N/A	Chemical staining and IF	ONH and TM	Collagen, elastin, Galectin and TGFB2RII	Whole tissue	↑ in elastin, collagen staining in the glaucomatous ONH. Elastin fibres showed thickening and curling in the LC collagen matrix. ↑ in Galectin and TGFB2II expression glaucomatous ONH, MTZ
Chauhan et al. 2019	12	11	70.3 ± 10.5	IHC, lipidomics	ONH	GBA, GBA2, ASAH1, and ASAH2	Whole tissue	↑ in glucosyl sphingosine in glaucoma and mirrored changes in GBA, GBA2, ASAH1, and ASAH2
Guan et al. 2022	27	19	75.8 ± 8.0	IHC	LC-cross sections	F-actin and GFAP	Astrocytes and Microglia	↓ GFAP and F-actin area fraction in glaucoma patients. ISH showed the presence of astrocytes and microglia
Neufeld 1999	11	6	63 ± 10	IHC	ONH	HLA-DR and CD45	Astrocytes and Microglia	Quiescent microglia were Stellate cells with thin, ramified processes, positive for HLA-DR and CD45 but GFAP negative, while activated cells were ameboid, larger, and clustered in compressed LC
Neufeld et al. 1997	12	14	74	IHC	ONH	NOS-1, NOS-2, and NOS-3	Astrocytes	NOS-1: prelaminar region, LC, and inside the diminished nerve fibre bundles. NOS-2: few cells in the disorganized LC. NOS-3: astrocytes and in the vascular endothelia of large and small vessels
Wang et al. 2002	10	7	87.1 ± 6.9	IHC	Retina	GFAP	Glial cells	Increased density of glial cells with deformed end feet in the peripapillary region. The glial cells were activated in the glaucomatous samples

Abbreviations: ISH: insitu hybridisation; RT-PCR: Reverse transcription Polymerase Chain Reaction, IHC: Immunohistochemistry, LC-MS/MS: Liquid-chromatography coupled mass-spectrometry, WB: Western Blot, IF: Immunofluorescence, ONH: optic nerve head, COX: cyclooxygenase, TNF: tumor necrosis factor, MMP: Matrix metalloproteinase, HSP: heat shock protein, TGF: transforming growth factor, NOS-2: nitric oxide synthase -2, c-fms: Colony-stimulating factor-1 receptor, TIMP: tissue inhibitor of metalloproteinases, TSP: thrombospondin, CD: Cluster of differentiation, PCNA: proliferating cell nuclear antigen, HLA-DR: human leukocyte antigen-DR subtype, HIF-1a: Hypoxia-inducible factor 1-alpha, MAPKs: Mitogen-activated protein kinases, AGEs: Advanced Glycation End Products, RAGE: receptor for advanced glycation end products, VEGF: Vascular endothelial growth factor, PDGF: Platelet-derived growth factor, TLR: Toll-like receptor, CFH: Complement factor H, AQP: Aquaporin, RhoA: Ras homolog family member A, ROCK: Rho-associated protein kinase, IgG: Immunoglobulin G, ANT3: ADP/ATP translocase 3, MeCp2: methyl-CpG-binding protein 2, DFS70: SRFS1-interacting protein 1, GBA: glucocerebrosidase, ASAH: acylsphingosine amidohydrolase, GFAP: Glial fibrillary acidic protein, LC: lamina cribrosa, RGC: retinal ganglion cells, NFL: Nerve fibre layer, IPL: inner plexiform layer

#### Inclusion and exclusion criteria

Studies were included in the review if they used human post-mortem samples of the retina and ONH to compare the changes in primary open-angle glaucoma with those from control and if they validated the molecular changes using immunohistochemistry or immunofluorescence techniques. Studies were excluded if they focused on any other types of glaucoma such as primary angle closure glaucoma, secondary glaucoma, congenital glaucoma, and pseudoexfoliation glaucoma, they used non-human samples for comparison, they had non-English text, or the full text was not available.

#### Data extraction

Extracted data included the number of patients, sex, age, the molecular technique used for visualisation, markers analysed, and the results of the comparison between the glaucoma patients and healthy controls. Quantitative data were extracted from the files provided or WebPlotDigitizer software (https://automeris.io/WebPlotDigitizer/) [4] was used to extract data from the graphs.

#### Data synthesis

The meta-analysis was performed using Review Manager statistical software (RevMan V.5, Copenhagen: The Nordic Cochrane Centre, The Cochrane Collaboration, 2014). The quantitative data were summarised by calculating standardized mean differences (SMDs) [5] with 95% CIs and the results were visualized as Forest plots. The risk of bias was assessed for the studies included in the review. GraphPad Prism and Excel were used for visualizing the graphs. Detailed information is provided in Supplementary File 1.

### RESULTS

A search of databases and a thorough manual literature review resulted in the identification of 91 relevant studies. Following additional scrutiny, 32 articles were selected for a comprehensive review, as they specifically compared glaucoma-induced alterations in post-mortem human retinal or optic nerve samples between control and glaucoma groups. The study flow diagram illustrates the screening and filtering criteria (Fig. 1). Details of the studies included, and the results have been summarised in Table 1. A meticulous examination of the included studies was undertaken, focusing on delineating the specific cell types associated with the molecular changes within the retina and optic nerve head (ONH). Among the cohort of 32 studies, 20 investigations ascribed the documented alterations to astrocytes (5 within the retina and 13 within the ONH), while 5 studies concentrated on the role of microglia. Additionally, 4 studies delved into diverse cell types, encompassing macrophages and Müller cells. Notably, 5 studies failed to establish a direct correlation between the observed changes in the retina and ONH with a specific glial cell type. A brief description of the risk of bias assessment has been provided in Supplementary File 1; the graph and summary are illustrated in Supplementary Figure 1.

**Table 2:** Cellular changes described in the reviewed studies.

Cell type	Tissue	Normal morphology	Changes to cell morphology	Studies
Astrocytes	Retina and ONH	Cells with smaller darker nuclei and fibrous processes	↑GFAP intensity	[8-10, 22, 28]
			Hypertrophy	[8, 10, 13, 16]
			Rounded cell morphology	[7, 8, 10, 13, 16]
			↑HLA-DR staining intensity	[18]
			Large flat cells with thick processes (advanced glaucoma)	[16]
			Cells with smaller, darker, and irregular nuclei placed in juxtaposition to blood vessels	[27]
Microglia	Retina and ONH	Small cell body with ramified processes	Amoeboid cells	[7, 29, 70]
			HLA-DR positivity	[70]
			Phagocytic cells with IgG deposition	[30]
Müller cells	Retina	Radial orientation of cells spanning all the retinal layers	Radial orientation spanning across all retinal layers and ↑GFAP expression	[23, 27]

### Influence of astrocyte activation on the ECM changes and immune response in glaucoma

**Figure 2.**
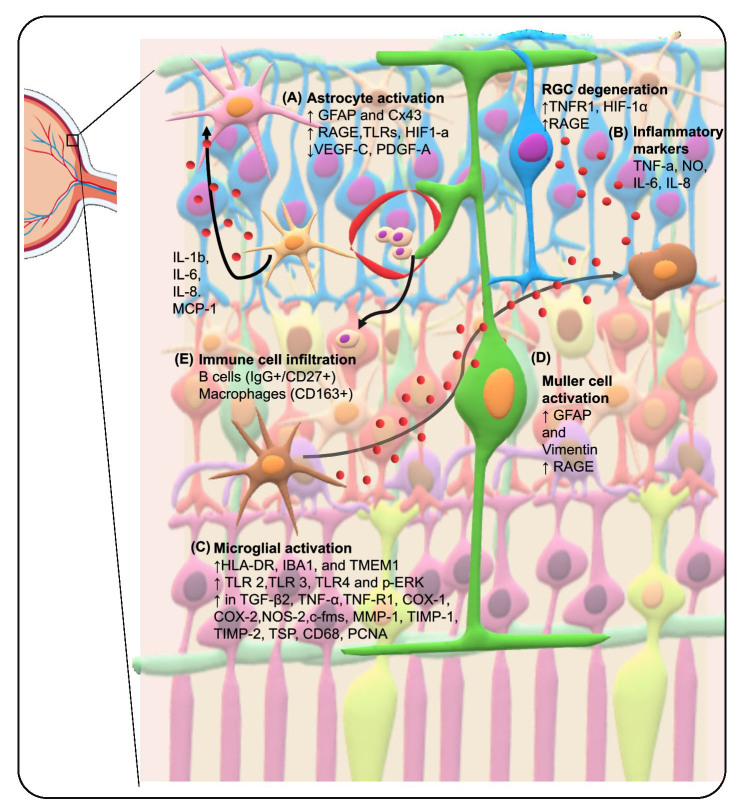
Schematic Overview of Molecular Alterations in Glaucomatous Tissues This figure provides a schematic representation of key molecular changes in glial cells as reported in the reviewed literature on human postmortem studies of glaucoma. A) Astrocyte Activation: Highlighted by increased GFAP expression and depicted by an elevated presence of Cx43, RAGE, TLRs, and HIF-1α. Additionally, activated astrocytes are shown producing pro-inflammatory cytokines, including TNF-α, IL-1b, IL-6, IL-8, and MCP-1. B) Inflammatory Marker Upregulation: An upsurge in TNFR and RAGE, alongside an increase in TNF-α and AGEs, suggests active glial cell interactions and their potential impact on retinal ganglion cell (RGC) death. C) Microglia Activation: Characterized by morphological changes and identified through Iba1 staining. A range of molecules, such as HLA-DR, Iba1, and TMEM119, are marked to signify dysregulation in activated microglia. D) Müller Cell Alteration: Illustrated by increased GFAP and vimentin expression, along with a rise in RAGE, reflecting a reactive gliosis. E) Immune Cell Infiltration: B cells (CD19+/CD27-) and macrophages (CD163+) infiltration are noted, indicating an immune response in glaucomatous tissues. Abbreviations: COX: cyclooxygenase, TNF: tumor necrosis factor, MMP: Matrix metalloproteinase, HSP: heat shock protein, TGF: transforming growth factor, NOS-2: nitric oxide synthase -2, c-fms: Colony-stimulating factor-1 receptor, TIMP: tissue inhibitor of metalloproteinases, TSP: thrombospondin, CD: Cluster of differentiation, PCNA: proliferating cell nuclear antigen, HLA-DR: human leukocyte antigen-DR subtype, IL: Interleukin, HIF-1a: Hypoxia-inducible factor 1-alpha, MAPKs: Mitogen-activated protein kinases, AGEs: Advanced Glycation End Products, RAGE: receptor for advanced glycation end products, VEGF: Vascular endothelial growth factor, PDGF: Platelet-derived growth factor, TLR: Toll-like receptor, CFH: Complement factor H, AQP: Aquaporin, TJs: Tight junctions

Of the 15 ONH studies, 12 localized the changes in the glaucomatous ONH to the astrocytes. The changes to the spatial arrangement and morphology of astrocytes in the retina and ONH have been summarised in Table 2. Both the glaucomatous ONH and retina showed increased Glial fibrillary acidic protein (GFAP) labelling indicating astrocyte activation. Astrocytes are known to provide structural support to the neurons. Indeed, an increase in extracellular matrix (ECM) proteins like collagen type IV (Fig. 2A) and Tenascin C has been reported in glaucomatous ONH[6-8]. The activated astrocytes in the glaucomatous eyes are reported to produce collagen type IV, which replaces the dying neurons [6, 7]. The 12 studies that reported changes to the ONH showed an increase in the anti-angiogenic factors (endostatin, collagen XVIII and ADAMTSL-3) [9], tight junction proteins (Connexin43) [10], the receptor for advanced glycation end products (RAGE)[11], endothelin B receptor (ETbR) [12], nitrotyrosine, nitric oxide synthase (NOS), tumour necrosis factor- α (TNF-α), heat shock protein 27 (HSP27) and cyclooxygenase-1 (COX-1) [13-17]. On the other hand, there was a decrease in the staining for pro-angiogenic factors [Vascular endothelial growth factor (VEGF)-C and platelet-derived growth factor (PDGF)-A](Fig. 2A and B)[9]. Lastly, some astrocytes in the ONH showed reactivity for human leukocyte antigen (HLA-DR) antibody suggesting that astrocytes in the glaucomatous ONH contribute to the immunogenic response [18]. Liquid chromatography coupled mass spectrometry (LC-MS/MS) analysis revealed toll-like receptor (TLR) signalling to be significantly dysregulated in the glaucomatous retina. In particular, the immunolabeling for TLR-3 was more prominent in astroglia [19]. Another LC-MS/MS analysis revealed that TNFR1 and several of its downstream interactors and kinases are upregulated in the glaucomatous retina. Nuclear factor-kappa light chain (NF-kB) subunits p65 and p50 were localized to GFAP-positive astrocytes. A similar increase was also observed for p-signal transducers and activators of transcription (STAT) in the RGCs and astrocytes. Moreover, TNFAIP3 labelling showed an increase in some glaucoma samples and was localized to RGCs and GFAP-positive astrocytes [20]. Immunostaining for the phosphorylated mitogen-activated protein kinases (MAPKs) such as extracellular signal-regulated kinases (ERK), c-Jun N-terminal kinase (JNK), and p38 showed that p-ERK staining was prominent and widespread and mostly colocalized with GFAP+ve astrocytes in the retina but was not exclusive to them [21]. In addition, complement factor H (CFH) was downregulated in the glaucomatous samples and some staining was localized to astrocytes [22].

We identified a few studies that used GFAP staining for localizing the molecular changes, however, they described the cells as glial cells. Nonetheless, the morphological assessment of the cells stained with GFAP indicates that these cells are astrocytes. One such study identified 3 types of glial cells, GFAP-positive astrocytes, Müller cells, and microglia. However, the description of glial cells was ambiguous, albeit they reported increased density of the glial cells in the peripapillary region with deformed end feet [23]. Other studies reported that increased expression of hypoxia-inducing factor (HIF)-1a[24], γ-Synuclein [25], matrix metalloproteinase (MMP)-1, MMP-2, MMP-3[26], TNF-α, and TNFR1[26, 27] was identified in glaucomatous ONH and retina. One study reported a decrease in aquaporin (AQP)-9 [28] staining with no change in the AQP-4 staining [28]. These findings indicate that the activated astrocytes are also involved in immune response. However, the exact interactions they have with the other cells are yet to be explored. Current evidence also suggests that these astrocytes might be involved in both pro- and anti-inflammatory processes depending upon the cues they receive from the environment.

### Drivers and activation patterns of microglia primary immune response in glaucoma

Microglia are the innate immune cells and first responders to pathogenic insults. These cells show a ramified morphology during the resting phase and become ameboid upon activation. Activated microglia were identified based on increased labelling for the cluster of differentiation (CD)-45, Iba1 or HLA-DR and amoeboid morphology. Cellular changes reported in the studies included are listed in Table 2. Yuan et al. observed that activated microglia in the glaucomatous ONH contain abundant TGF-β2, TNF-α, and proliferative cell nuclear antigen (PCNA). The overall staining suggests that COX-2 expression was differential and dependent on specific disease processes. Few microglia from the prelaminar to the post-laminar regions stained for TGF-β1, NOS-2, thrombospondin, tissue inhibitor of matrix metallo-proteinase (TIMP)-2, and CD68 in glaucomatous tissue. MMP-1, MMP-2, MMP-3, MMP-14 COX-1, TNF-R1, TIMP-1, and c-fms labelling increased in microglia in the glaucomatous ONHs. Whereas HSP27 was not present in microglia[29]. Luo et al showed that microglia stained prominently for TLR 2, TLR3, and TLR4[19](Fig. 2C). Staining for p-ERK and phosphor-p38 was prominent and widespread, and the staining was not exclusive to HLA-DR-positive microglial cells[21]. There is enough evidence regarding the microglial involvement in the immune responses. Like the astrocytes, even the microglia are activated based on the environmental stimuli. Further, these microglia may present diverse phenotypes that influence the trajectory of the disease. Therefore, understanding the diverse nature of microglia is important to understand its implication in glaucoma progression.

### Responses in other cells supplement the glial cell responses

**Figure 3.**
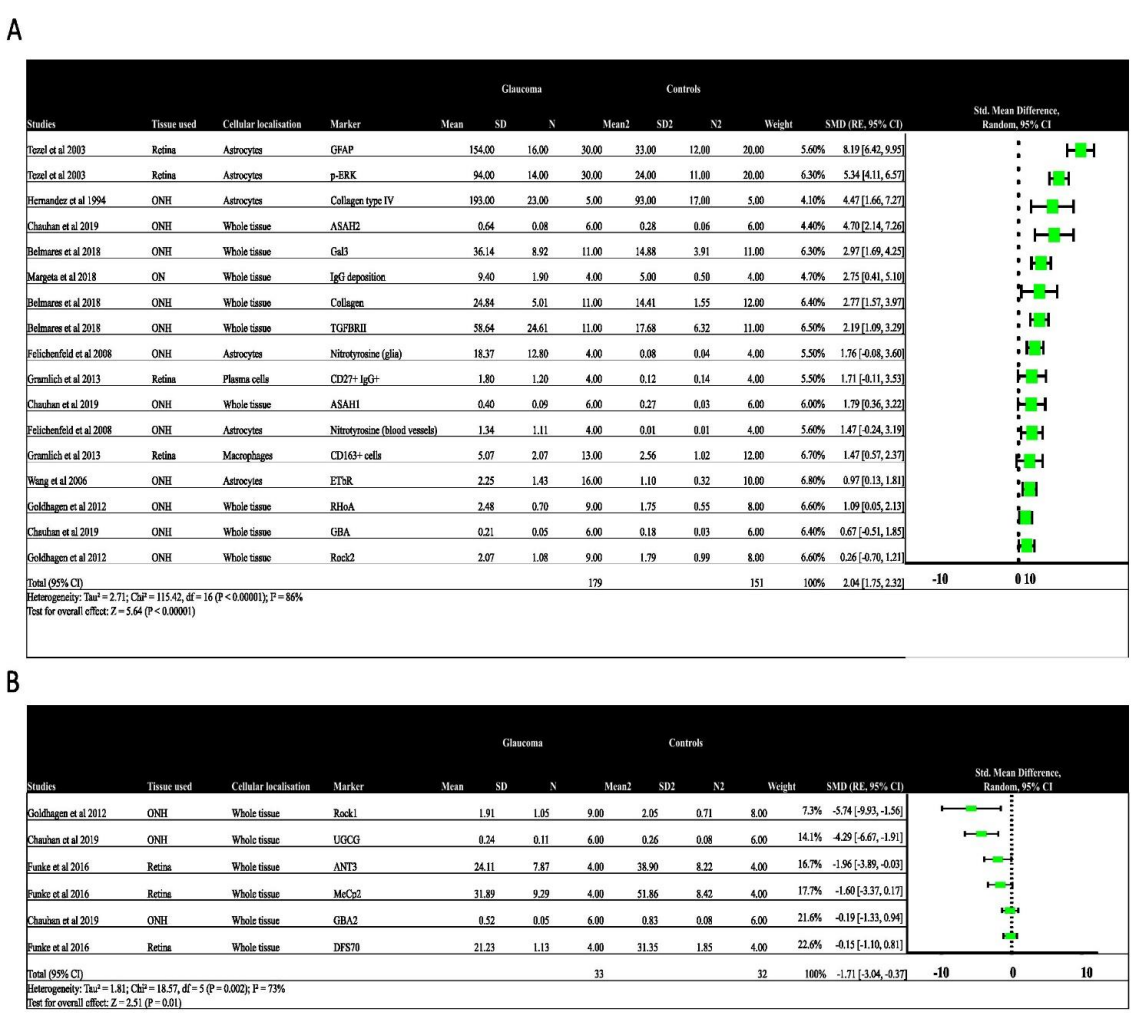
Forest plot showing the quantitative data from the ten studies. A) molecular changes positively associated with neuroinflammation and B) molecular changes negatively associated with neuroinflammation. Abbreviations: ONH: optic nerve head, CD: Cluster of differentiation, RhoA: Ras homolog family member A, ROCK: Rho-associated protein kinase, IgG: Immunoglobulin G, ANT3: ADP/ATP translocase 3, MeCp2: methyl-CpG-binding protein 2, DFS70: SRFS1-interacting protein 1, GBA: glucocerebrosidase, ASAH: acylsphingosine amidohydrolase, GFAP: Glial fibrillary acidic protein, ETbR: Endothelin receptor B.

Apart from the astrocytes and microglia, Müller cells are also involved in glaucoma pathogenesis. However, only two studies discussed the changes in the Müller cells. The Müller cells were characterized by GFAP staining across all the layers of the retina and showed increased connexin43. These cells seemed activated based on morphological assessment [10]. Increased RAGE labelling was also observed in these radially oriented, vimentin-positive Müller cells [11] (Fig. 2D). Further, infiltrating cells could enter the retina through a leaky blood-retina barrier and potentially influence glaucoma pathology. To this end, Gramlich et al reported an increase in the levels of pro-inflammatory cytokines such as TNF-α, interferon (IFN)-γ, interleukin (IL)-1β, IL-6, and IL-8 (Fig. 3B) in the retinal tissue using microarray. They even observed an increased accumulation of IgG antibodies and CD27^+^/IgG^+^-plasma cells in the glaucomatous retina. However, they could not confirm T-cell presence due to a lack of CD27^+^/CD3^+^ cells [30]. Another study reported an increase in CD163+ macrophages in axon bundles in ONH in glaucomatous samples [31] (Fig. 2E). Glaucoma pathology is characterized by the loss of RGCs. Therefore, looking for morphological changes in the RGC is pertinent. Indeed, there was a decrease in CFH and a more prominent decrease in NeuN and BRn3a-positive RGCs. However, there were no significant changes in the staining for complement regulators such as CD35 and CD59, in glaucomatous samples [22]. In addition, there was an increase in HIF-a staining in the optic nerve axons and RGCs in glaucoma patients [32]. Four studies reported the molecular changes in the glaucomatous retina and ONH; however, they did not associate the changes to a certain cell type. Belmares et al reported increased staining for ECM proteins like galectin, collagen, and TGFβ2RII in the ONH and the increase in TGF-Β 2RII correlated with galectin [33]. There was an increase in the Ras homolog family member (RhoA) and Rho-associated protein kinase (ROCK)-2 (not significant) staining in the glaucomatous ONH but a decrease in the staining for ROCK-1 [34] (Fig. 3A and B). Lastly, two studies looked at the changes in the lipidome and proteome in the ONH and retina, respectively. Lipidomics revealed a significant increase in glucosylsphingosine in the glaucomatous retina and change was also seen in the lysosomal and non-lysosomal enzymes involved in the glucosylsphingosine metabolism pathway. Increase in levels of acylsphingosine amidohydrolase 1(ASAH1) and ASAH2, whereas a decrease in glucocerebrosidase 2 (GBA2) and no changes in GBA and UGCG glaucomatous samples [35]. On LC-MS/MS analysis, Funke et al [36] reported that there was a decrease in mitochondrial proteins such as ADP/ATP translocase 3 (ANT3), cytochrome C oxidase subunit 7A2 (COX7A2) and pyruvate dehydrogenase component subunit ß (PDHE1-B), retinal nuclear proteins like methyl-CpG-binding protein 2 (MeCp2) and SRFS1-interacting protein 1 (DFS70). There was an increase in stress-related proteins such as serotransferrin, crystallins, and glutathione metabolic proteins. However, they could only validate the downregulation of ANT3, MeCp2 and DFS70. These findings indicate that glaucoma progression is influenced by the different interactions between the glial cells. Therefore, emphasizing the need for further exploration of these interactions. In addition, these studies also highlight the importance of understanding the role of interactions across the BRB. This will aid in understanding the role of systemic immune cells in glaucoma progression.

### Quantitative analysis of molecular markers

Among the studies included, ten reported quantitative data regarding the observed changes. Standardised mean differences (SMDs) are used as a summary statistic when comparing studies assessing the same outcome using different ways of measurement. We used the SMDs to understand how the molecular changes reported in the studies included in the review influence neuro-inflammation in glaucoma. The molecular changes associating positively with neuroinflammation (I^2^ = 78%; effect size = 2.04 SMDs; 95% CI =1.75-2.32) (Figure 3a) were plotted together, whereas those that did not favour neuroinflammation in glaucoma were plotted together (I^2^ = 73%; effect size = -0.90; 95% C.I = -1.51- -0.29) (Fig. 3B). Indeed, we observed that molecular changes that were associated positively with neuroinflammation like GFAP, p-ERK, nitrotyrosine, ETbR and RHoA showed upregulation in glaucomatous tissues. The remaining studies have described their observations without any quantification. Unlike in the brain, there is a lack of studies correlating molecular changes with glial cell activation in the human retina and ONH of glaucoma. This lack of comprehensive studies impedes meta-analysis due to variability in the samples used, patient-to-patient variability and lack of uniform clinical details.

### DISCUSSION

In this systematic review, we analysed molecular changes reported in the retina and ONH using post-mortem human samples. Most of the scrutinized studies delineated instances of glial cell activation, concurrently linking such activation to pro-inflammatory changes. These findings imply a consequential impact of neuroinflammation on the fate of retinal ganglion cells (RGCs). Nevertheless, the precise mechanistic underpinnings governing the loss of RGCs remain elusive. Additionally, it is noteworthy that the pathogenesis of glaucoma exhibits shared features with other neurodegenerative pathologies, such as amyotrophic lateral sclerosis (ALS), Alzheimer's disease (AD) [37], Parkinson's disease [38], Huntington's disease, and frontotemporal dementia. Notably, these pathologies have been associated with neuroinflammatory processes. Hence, a comprehensive comprehension of the intricate interactions among distinct glial cell subtypes, RGCs, and systemic immune cells assumes significance.

Microglia represent the principal immunocompetent cells within the retina, eliciting activation in response to injurious or stress-inducing stimuli. These cells demonstrate prompt migratory responses to the loci of injury, thereby substantively participating in neuroinflammatory processes. Conversely, macroglia cells, encompassing astrocytes and Muller cells, assume a supportive role. In the context of injury or stress, these cells exhibit mobilization to facilitate the redistribution of resources. Consequently, concerted interactions between microglia and macroglia cells are imperative for the modulation of neuroinflammatory responses [39]. In our review, of the 32 studies, 27 included this review reported activation of glial cells such as microglia, astrocytes, or Müller cells (Fig. 3A). Furthermore, the molecular alterations were found to be concurrently associated with multiple types of glial cells. This observation substantiates the notion that interactions among glial cells in the retina play a pivotal role in mediating inflammatory responses and, subsequently, contribute to the progression of glaucoma. The glial cells can work together as they share receptors for inflammatory responses like TLRs. Further, the glial cells could interact with each other to regulate the shared responses [40]. Lastly, glial cells might relay signals to other cell types and amplify [41, 42] or regulate [43] inflammation or neurodegeneration. Some of these interactions have been reported using animal models. A mouse model-based study has reported that interaction between Muller cells and microglia aggravates the inflammatory responses [44]. They reported an increase in the pro-inflammatory factors in Müller cells in response to microglial activation.

Even among the studies included in our review, we could identify some hallmarks of classic inflammatory responses in the glaucomatous retina and ONH. There was an increase in the Toll-like receptor (TLR) with increased activity of glial cells. TLR 3 was prominently associated with astrocytes. Coincidently, there was an increase in damage-associated molecular patterns (DAMPS) such as heat shock proteins (HSPs). The DAMPS are endogenous molecules that are ligands for the TLRs and perpetuate an inflammatory response. An increase in HSP staining has been reported in glaucomatous ONH[17] and retina [19]. Other DAMPS such as ATP could also induce inflammasome formation via the pyrogenic channels[45-47]. These findings, however, have not been validated using human samples. Moreover, Tenascin-C, an ECM glycoprotein, was also associated with glaucoma[8] and was further reported to support proinflammatory responses using the TLR4 receptor. The TLRs then recruit other proteins downstream, which then induce the activation of MAPKs and downstream kinases. This leads to the production of downstream amplifiers and effectors. Indeed, an increase in MAPKs has also been observed in the glaucomatous samples. Phospho-ERK was more prominent in microglia and astrocytes in glaucoma. The widespread activation of ERK indicates its role in glial cell activation. This shows that the DAMPs associated pathways are activated in both astrocytes and microglia and the activation persists even after initial insult. In addition, there was an increase in the TNF-α expression in the glaucomatous retina and ONH. This increase was associated with both astrocytes and microglia. There was also an increase in the IFN-γ, IL-1β, IL-6, and IL-8 levels in the retina. This was parallel to an increase in the TNFR1 levels in the RGCs, indicating the susceptibility of RGCs to the TNF-α induced cell cytotoxicity. TNF-α could also induce indirect cell death by inducing nitric oxide and endothelin-1 synthesis, activating MMPs, and modulating excitotoxic injury [48]. Similarly, one of the LC-MS/MS analyses using human retinal samples also showed co-activation of different pathways like the caspase- and calpain-mediated pathways, mitochondrial dysfunction, and ER stress. On the other hand, there was an increase in TGF-β2 and COX-1 in the glaucomatous retina and ONH. TGF-βs have a function opposite to that of TNF-α in terms of the immune response. TGF-β production in activated glia in the glaucoma samples suggests upregulation of neuroprotective response [29]. Further, COX-1 has also been reported to downregulate the cytotoxic microglia [13, 29]. This indicates that the glial cells may be involved in both pro and anti-inflammatory responses based on the microenvironment (Fig. 3C). However, the existing literature using animal models supports the claim that during early disease, the glial cells perform neuroprotective effects, but persistent insults might drive the glial cells to produce pro-inflammatory markers and contribute detrimentally to neuro-destruction. Nonetheless, the review also suggests that in humans glial cells with different functional states showing diverse phenotypes may be present. Further, this diversity in the phenotype and function might be due to the interactions among them or in response to environmental stimuli. Therefore, an increased understanding of these glial interactions could be helpful in the early prognosis of glaucoma or in developing new immune-modulatory therapies.

We still have a limited understanding of the correlation between glial cell reactions and the clinical trajectory of the disease. Very little is known about the glial cell phenotype in human glaucoma. Nonetheless, glial cells are reported to show multiple phenotypes that influence glaucoma progression. Microglia are phagocytic cells that usually adapt to the amoeboid morphology after extensive damage, which helps in chemotactic motility toward the site of injury [49]. However, a few other phenotypes such as rod-like microglia with narrow and elongated cells with scanty cytoplasm and few processes have been reported in mouse models of glaucoma. These rod-like microglia have been associated with retinal neurodegeneration[50]. Another microglial phenotype is the disease-associated microglia (DAM) which shows an increase in the upregulation of genes involved in lysosomal, phagocytic, and lipid metabolism pathways [51]. Transcriptome analysis of adult retina from a transgenic mouse model of glaucoma showed DAMs and their profile was similar to that in the brain [52]. However, no studies have reported the prevalence of these phenotypes in the human retina. Even the studies included in the review lacked comprehensive analysis correlating the morphological and functional changes in the glial cells. The phenotypic variation in microglia could drive the pathogenesis of glaucoma (Fig. 3B). Similarly, astrocytes are another major glial cell type that has been reported to show regional heterogeneity indicating differential regional responses to glaucoma. Astrocytes have been reported to have two phenotypes: A1 and A2. In glaucoma, the astrocytes can be responsible for neurodegeneration and protection by acquiring A1 and A2 phenotypes, respectively [53]. In neurodegenerative phenotypes, there is an increase in factors like Il-1α, TNF, and C1q. Human studies have also shown an increase in the TNF-α labelling in association with activated astrocytes. On the other hand, anti-inflammatory markers like STAT-3 have mediated neuroprotective functions. One study in this review showed an increase in p-STAT3 in glaucomatous samples and the staining was associated with activated astrocytes [20]. This might indicate the presence of a continuum of phenotypes with divergent functions rather than just A1 and A2 astrocytes. Astrocytes are important for normal ONH and retina activity, homeostasis maintenance, neuronal activity regulation, and immune responses. In the ONH, astrocytes play a major role in inducing changes to the architecture by mediating ECM remodelling and causing ONH cupping [54]. Further, it has been reported that microglia could modulate the astrocyte to show a neurotoxic phenotype. Also, there have been animal model studies reporting how astrocyte phenotype could regulate the microglial immune responses [41, 55]. Therefore, understanding the prevalence of glial cell phenotypes becomes crucial for developing newer prognostic markers or potential immunomodulatory therapies.

Another glial cell type, Müller cells, are radial glial cells that span the whole retina and surround the retinal neurons. They are also involved in maintaining the blood-retinal barrier. They have been known to be involved in the retinal metabolism of glucose and neurotransmitters [56]. Evidence reported so far emphasizes the role of Müller cells in RGC survival and interactions with other glial cells [57, 58]. Nonetheless, studies observing the role of Müller cells using human samples are lacking.

Interactions with systemic immune cells also affect glaucoma progression. However, only two studies have so far reported the presence of systemic immune cells such as plasma (IgG+/CD27+)[30] cells and CD163+ [31] macrophages in the retina. In addition, these studies could not observe the infiltration of macrophages or other types of T cells. Nonetheless, studies using glaucoma models have reported macrophage infiltration as a critical step for glaucoma pathogenesis [52, 59]. This evidence and those reported using animal models suggest that the activated resident glial cells might send signals that recruit systemic immune cells to the retina. However, the retina is an immune privilege site. Therefore, immune infiltration of systemic cells indicates the disruption of the blood-retinal barrier (BRB). Consequently, it has been observed that there is an increase in cell-adhesion molecules on the endothelial cells in inflamed retinas. Moreover, RGCs have been reported to express many chemokines that are responsible for chemokine recruitment [60-62]. However, many aspects of immune infiltration and BRB integrity and their role in glaucoma pathogenesis remain elusive (Fig. 3D). This necessitates further studies focusing on the interactions across the blood-retinal barrier for an increased understanding of the role of systemic immune cells in glaucoma pathogenesis.

**Figure 4.**
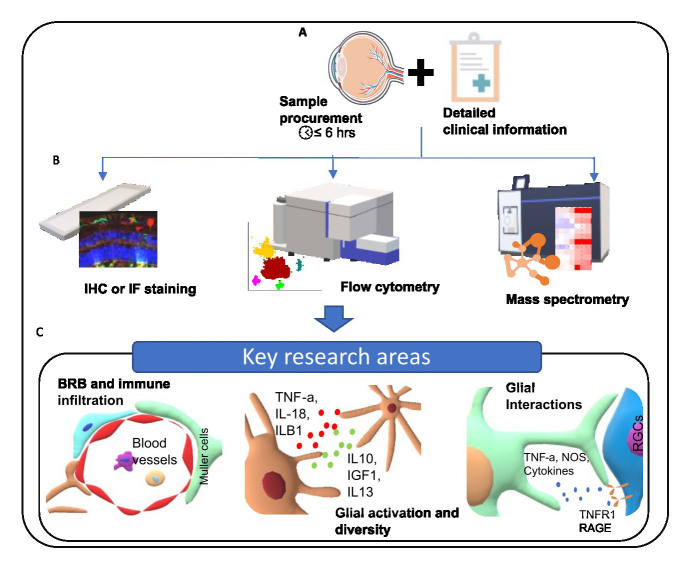
Proposed Framework for Bridging Research Gaps in Glaucoma Studies. This figure outlines a hypothetical experimental plan designed to address existing gaps in understanding the roles of glial changes and neuroinflammation in glaucoma research. A) Streamlined Sample Acquisition: Ensures the integrity of samples and the immediate availability of comprehensive clinical data. B) Advanced Analytical Techniques: Depicts the use of high-resolution, high-throughput methodologies such as immunohistochemistry (IHC), flow cytometry, and mass spectrometry, aiming to produce robust and reliable evidence to enrich our understanding of neuroinflammation in glaucoma. C) Key Research Domains: Highlights critical areas for future research, including the interactions at the blood-retinal barrier, the diversity and function of glial cells, and their communication networks.

The existing literature provides evidence to associate increased activation of glial cells in glaucoma with dysregulation of the immune response, homeostatic components of the cells and increased antigen presentation. This indicates that neuroinflammatory responses are driven at least in part by a complex interplay of different pathways and interactions between different cell types. These responses are very dynamic and necessitate further studies to disseminate spatial and temporal changes in glial cell responses throughout the trajectory of the disease. Increased understanding of these interactions would aid in stratifying patients who are prone to progress to blindness and administering preventive therapies for better prognosis. In addition, this knowledge can be very crucial while developing immunomodulatory therapies for managing glaucoma beyond the alleviation of IOP. Although recent experimental studies have very successfully reported immune modulation for glaucoma. Clinical translation is challenging due to the lack of knowledge about the complexity of cellular and molecular components of neuroinflammation [63]. However, while several studies identify key cellular responses, there are still gaps in the current knowledge regarding immune mechanisms in glaucoma and interactions between glial cells and neuronal cells. This knowledge gap, in part, stems from the scarcity of studies using human samples, with a predominant focus on research conducted using animal or in-vitro models of glaucoma. These models do not accurately duplicate human glaucoma and there are several tissue-level differences such as the lack of LC in mice. We recognize the challenges associated with obtaining and managing human post-mortem samples. These challenges include the limited accessibility of donor tissues and acquiring human post-mortem samples a formidable task. Additionally, the procedural complexities involved in obtaining ethical approval, securing consent from donors or their legal representatives, and preserving tissue viability pose significant challenges. Moreover, the variable progression rates among patients in the cohort underscore the importance of meticulous attention to patient identity in the research process. Further, the usual timeline of acquiring the post-mortem samples renders them non-viable for many molecular analyses like the flow-cytometry. Nonetheless, there have been studies that have used post-mortem tissue to assess healthy human retinas at single-cell resolution [64, 65]. However, such studies for assessing glaucoma-related changes using transcriptomics approaches have been limited to animal models [66-69]. Most of the existing studies using post-mortem human tissues have used a reductionist approach, whereby they have focused on a single target. These studies have not focused on neuroinflammatory responses specific to glial cell subtypes. More systemic approaches correlating the changes among glial cells and RGCs should be undertaken. This would provide us with more robust data and an increased understanding about the influence of glial cell response on glaucoma progression. In particular, we identify three major areas that need to be focused with respect to glaucoma pathogenesis. 1) Interactions at the blood-retinal barrier to understand the role of systemic immune system in glaucoma prognosis. 2) Glial cell activity and diversity and correlate it to glaucoma progression. 3) Profile glial cell interactions to understand how they influence the disease progression. Therefore, we highlight the need for streamlined sample acquisition with detailed clinical information regarding glaucoma diagnosis. This will ensure maintenance of sample integrity with sufficient clinical information to correlate the findings. Further, the use of high-resolution, high-throughput multi-omic approaches would help in building a robust body of evidences to address the research gaps in glaucoma pathogenesis. We have visualised a proposed framework for bridging the research gap in Figure 4. By addressing these research gaps, the field can potentially advance towards more effective prognostic markers and immunomodulatory therapies for managing glaucoma beyond the traditional focus on intraocular pressure.

### Conclusion

In conclusion, this review delves into the available literature to explore existing evidence of dysregulation of inflammatory responses in glaucoma. While significant strides have been made in identifying key cellular responses, the existing literature reveals notable gaps in our understanding of immune mechanisms and the nuanced interactions between glial and neuronal cells. A predominant reliance on animal and in-vitro models, lacking crucial human-specific features like the lamina cribrosa, highlights the need for more comprehensive investigations utilizing human samples. In moving forward, addressing these knowledge gaps requires a concerted effort to overcome challenges associated with human sample acquisition and management. Therefore, more extensive, systematic studies, particularly those incorporating post-mortem human tissues, should be undertaken to increase our understanding of the role of immune mechanisms in glaucoma. Such endeavours will undoubtedly pave the way for innovative therapeutic strategies and a more holistic approach to managing glaucoma.

## Supplementary Materials


