# Supplementary material for: Glial Cell Activation and Immune Responses in Glaucoma: A Systematic Review of Human Postmortem Studies of the Retina and Optic Nerve
Source: Aging Dis. 2024 Oct 1;15(5):2069–83. doi: 10.14336/AD.2024.0103 (PMC11346413; doi:10.14336/AD.2024.0103)
Supplement: Supplementary file 1 [file AD-15-5-2069-s.pdf]

SUPPLEMENTARY DATA

Glial Cell Activation and Immune Responses in Glaucoma: A Systematic Review of Human Postmortem Studies of The Retina and Optic Nerve

Akanksha Salkar, Roshana Vander Wall, Devaraj Basavarajappa, Nitin Chitranshi, Gabriella E. Parilla, Mehdi Mirzaei, Peng Yan, Stuart Graham, Yuyi You

© 2024. Salkar A et al. Published online at http://www.aginganddisease.org/EN/10.14336/AD.2024.0103

SUPPLEMENTARY DATA

1. Systematic Search Protocol

Written based on the WHO Review Protocol Template, 2011

1. Title: Neuroinflammatory changes in post-mortem glaucoma samples: a systematic review

2. Background: There is a dearth of studies assessing changes in the human retina and optic nerve. There is no summarized information about the existing literature pertaining to changes in the retina or optic nerve in glaucoma patients.

3. Objective: The research aims at collating the existing literature related to changes in the retina or optic nerve in patients with glaucoma using post-mortem samples.

4. Review Question (Population Intervention Comparison Outcome)

Population: Post-mortem samples from patients with a confirmed glaucoma diagnosis

Intervention: N/A

Comparison: Normal controls

Outcome: Changes in the glaucomatous retina and optic nerve in glaucoma patients.

5. Evidence Gathering and Study Selection (list databases, websites, and journals that will be searched)

Ovid will be used to query the Medline and Embase databases together using exp (MeSH) terms and title, abstract, and keywords search terms. Full search terms and results found are listed in the Appendix.

Reference searches: Bibliographies of papers deemed eligible for this review will be hand searched to identify any additional eligible references, which will then be screened for title, abstract, or full text as appropriate.

6. Eligibility Criteria

The results of these searches will be combined and deduplicated using Ovid. They will then be screened for title and abstract, and then full text using the following eligibility criteria.

i) Type of study included: Papers published in peer-reviewed journals.

ii) Types of participants: Studies that include post-mortem retina or optic nerve samples from patients with glaucoma will be included. There should be a confirmed diagnosis of glaucoma before the autopsy. Controls should be non-glaucomatous samples confirmed at autopsy.

iii) Types of outcome measures: The studies should assess molecular changes in the retina and optic nerve of patients with glaucoma. The study should report the chnage

7. Exclusion Criteria

Reviews, animal studies, and studies with no confirmed diagnosis of glaucoma will be excluded. Data published as conference proceedings and abstracts will be excluded. Studies will not be included to measure anatomical changes to the retina or optic nerve in living subjects. Lastly, studies with glaucoma types other than primary open-angle glaucoma (POAG) such as primary angle closure glaucoma, congenital or pseudoexfoliative glaucoma and those without normal control will be excluded.

8. Data extraction

Data extracted will include:

• Citation information
• Origin of eye tissue (hospital, brain bank etc.)
• Method of characterization of glaucoma cases and controls
• Subject age, sex and ethnicity, duration in a fixative solution, region examined, method of measuring used, and results. The final decision on what to include in the published tables will be at the authors' discretion based on the importance and variability within the studies.

9. Data Synthesis

The changes to the retina or optic nerve using post-mortem human samples will be collated as appropriate based on the number of papers. The quantitative data will be used to calculate the standardized mean difference and risk of bias among on studies included.

10. Dissemination

The data will be used for preparing a manuscript and submitting it to an ophthalmology journal.

2. Search query

Database used:

1. Embase <1974 to 2023 March 06>

© 2024. Salkar A et al. Published online at http://www.aginganddisease.org/EN/10.14336/AD.2024.0103

SUPPLEMENTARY DATA

2. Ovid MEDLINE(R) ALL <1946 to March 06, 2023>

Date of search: March 08, 2023

Query No.	Query	Number of papers
**1**	(((((glaucoma or Glaucomas or glaucomatous) and retina) or optic nerve) and histology) or immunohistochemistry or molecular changes).mp. [mp=ti, ab, hw, tn, ot, dm, mf, dv, kf, fx, dq, bt, nm, ox, px, rx, ui, sy, ux, mx]	1286421
**2**	glaucoma.m_titl.	82578
**3**	(retina or optic nerve).m_titl.	72512
**4**	limit 3 to article	63240
**5**	2 and 4	1724
**6**	limit 5 to english language	1464
**7**	((glaucoma and markers) or changes or effects on retina or optic nerve).ab.	5154177
**8**	6 and 7	1149
**9**	((molecular changes or proteins or human samples or patients or retina or optic nerve) and glaucoma).ab.	85081
**10**	8 and 9	1093
**11**	(embedded or immunohistochemistry or immunolabeling or protein or DNA or RNA).mp. [mp=ti, ab, hw, tn, ot, dm, mf, dv, kf, fx, dq, bt, nm, ox, px, rx, ui, sy, ux, mx]	14807501
**12**	10 and 11	186
**13**	limit 12 to human	75
**14**	limit 13 to humans	75
**15**	remove duplicates from 14	46

3. Data Synthesis

Meta-analysis was performed by comparing glaucoma's molecular changes to control samples by calculating the standardized mean differences (SMDs). The sample mean, standard deviation and the number of samples were used to calculate the SMD. The SMD was calculated using the random-effects model using inverse variance method as we expected heterogeneity among the studies. I^2 statistic was used to determine heterogeneity. Data are presented as SMD [95% confidence interval] in Figure 2.

4. Risk of bias assessment

The quality assessment was performed for all the 32 studies included in the review. Four potential points that would introduce bias were considered:1) whether the assessment was blinded 2) whether controls included were age-matched, 3) lacking information regarding the patient status and 3) gaps in data reported. The risk of bias was assessed using four parameters: Lack of clinical details or patient demographics (selection bias), matching for age (selection bias), blinded assessment (performance bias), and gap in reported quantitative data (reporting bias). Most of the bias was attributed to the gap in reported quantitative data. Of the 32 studies, 17 studies provided a detailed description of clinical characteristics. In addition, 8 studies did not provide information on age-matched controls and 13 studies did not provide any information regarding the blinded assessment. In general, the studies included in the review were of good quality. The risk of bias summary and graph for all the studies are provided in Supplementary Figure 1.

© 2024. Salkar A et al. Published online at http://www.aginganddisease.org/EN/10.14336/AD.2024.0103

SUPPLEMENTARY DATA

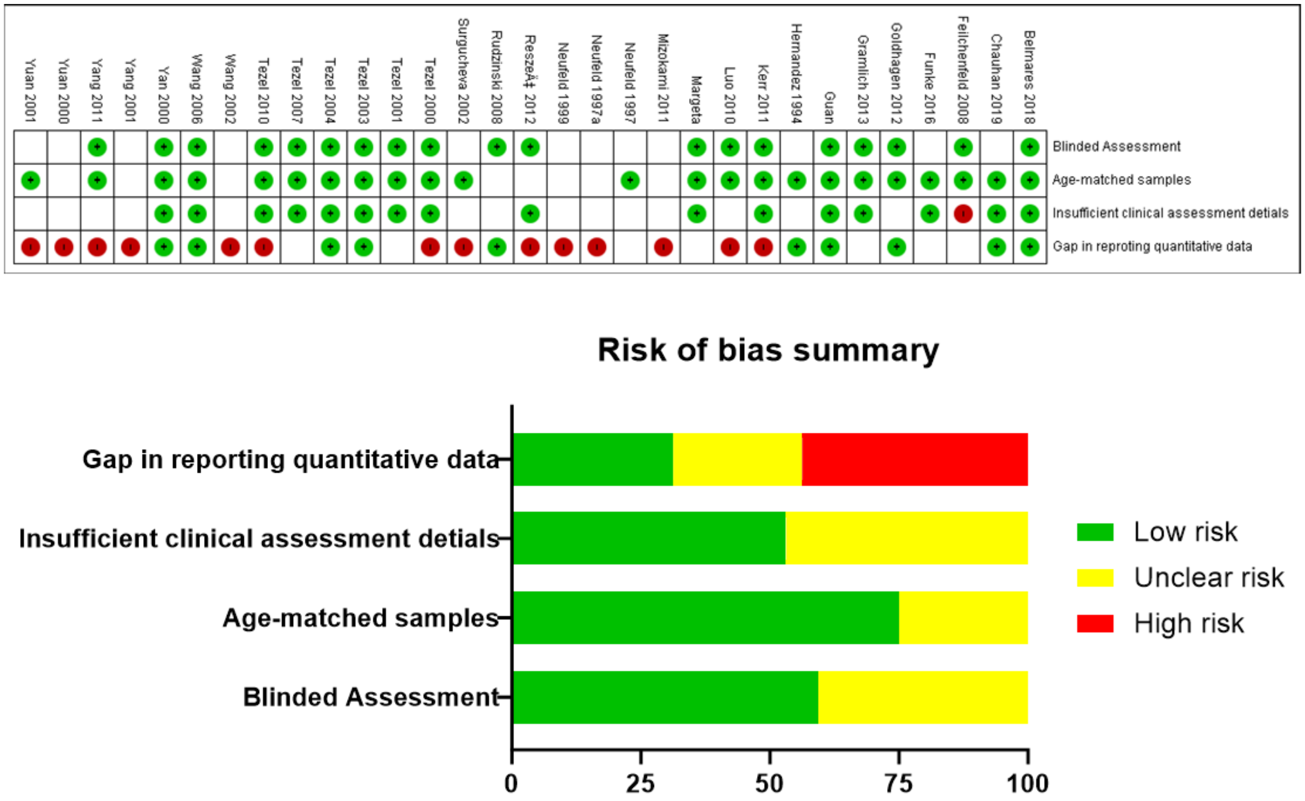

Supplementary Figure 1. Risk of bias analysis for studies included in the review. A) risk of bias summary B) Risk of bias graph
